# Intrinsic Programming of Alveolar Macrophages for Protective Antifungal Innate Immunity Against *Pneumocystis* Infection

**DOI:** 10.3389/fimmu.2018.02131

**Published:** 2018-09-19

**Authors:** Samir P. Bhagwat, Francis Gigliotti, Jing Wang, Zhengdong Wang, Robert H. Notter, Patrick S. Murphy, Fátima Rivera-Escalera, Jane Malone, Michael B. Jordan, Michael R. Elliott, Terry W. Wright

**Affiliations:** ^1^Department of Pediatrics, University of Rochester School of Medicine and Dentistry, Rochester, NY, United States; ^2^Department of Microbiology and Immunology, University of Rochester School of Medicine and Dentistry, Rochester, NY, United States; ^3^David H. Smith Center for Vaccine Biology and Immunology, University of Rochester School of Medicine and Dentistry, Rochester, NY, United States; ^4^Divisions of Immunobiology, and Bone Marrow Transplantation and Immune Deficiency, Cincinnati Children's Hospital Medical Center, University of Cincinnati College of Medicine, Cincinnati, OH, United States

**Keywords:** innate immunity, fungal pathogens, pneumocystis, mouse models, alveolar macrophage

## Abstract

Invasive fungal infections, including *Pneumocystis* Pneumonia (PcP), remain frequent life-threatening conditions of patients with adaptive immune defects. While innate immunity helps control pathogen growth early during infection, it is typically not sufficient for complete protection against *Pneumocystis* and other human fungal pathogens. Alveolar macrophages (AM) possess pattern recognition molecules capable of recognizing antigenic and structural determinants of *Pneumocystis*. However, this pathogen effectively evades innate immunity to infect both immunocompetent and immunosuppressed hosts, albeit with differing outcomes. During our studies of mouse models of PcP, the FVB/N strain was identified as unique because of its ability to mount a protective innate immune response against *Pneumocystis* infection. In contrast to other immunocompetent strains, which become transiently infected prior to the onset of adaptive immunity, FVB/N mice rapidly eradicated *Pneumocystis* before an adaptive immune response was triggered. Furthermore, FVB/N mice remained highly resistant to infection even in the absence of functional T cells. The effector mechanism of innate protection required the action of functional alveolar macrophages, and the adoptive transfer of resistant FVB/N AMs, but not susceptible CB.17 AMs, conferred protection to immunodeficient mice. Macrophage IFNγ receptor signaling was not required for innate resistance, and FVB/N macrophages were found to display markers of alternative activation. IFNγ reprogrammed resistant FVB/N macrophages to a permissive M1 biased phenotype through a mechanism that required direct activation of the macrophage IFNγR. These results demonstrate that appropriately programmed macrophages provide protective innate immunity against this opportunistic fungal pathogen, and suggest that modulating macrophage function may represent a feasible therapeutic strategy to enhance antifungal host defense. The identification of resistant and susceptible macrophages provides a novel platform to study not only the mechanisms of macrophage-mediated antifungal defense, but also the mechanisms by which *Pneumocystis* evades innate immunity.

## Introduction

*Pneumocystis* (Pc) is an opportunistic fungal pathogen that causes life-threatening pneumonia (PcP) in immunocompromised individuals, including patients with AIDS, cancer, organ transplants, and certain congenital immunodeficiencies. The species infecting humans, called *P. jirovecii* (Pj), is widespread throughout the human population. PcP remains one of the most common AIDS-defining-illnesses (1, 2), and in recent years non-AIDS-related PcP has become more frequently diagnosed in previously unreported clinical settings (3). Patients receiving long-term immunomodulatory therapies for inflammatory disease may be at particular risk. Persistent colonization with *P. jirovecii* has also been reported, and is a complicating factor in patients suffering from chronic lung disease (4). Despite improvements in medical care, the mortality rate for patients developing PcP has changed little over the past three decades, and greater understanding of host-pathogen interactions is critical to improving treatment and survival.

*Pneumocystis* appears ubiquitous throughout the human population. Over 80% of normal children harbor anti-Pc antibodies by two years of age, suggesting that they have been exposed to or infected with this pathogen (5). There have also been more frequent reports of *Pneumocystis* in non-immunosuppressed patients (6, 7), and studies using animal models have confirmed that immunocompetent hosts become transiently infected prior to the onset of adaptive immunity (8, 9). Furthermore, mice with functional innate immunity but defective adaptive immunity (SCID, Rag, Nude, CD4^+^ T cell-depleted) are highly susceptible to PcP. Thus, *Pneumocystis* must possess the capability to evade the early pulmonary innate immune response. Alveolar macrophages constitute a major component of innate immunity in the lung. These sentinel cells express pattern recognition receptors capable of recognizing antigenic and structural components of *Pneumocystis* (10), but for reasons that remain unclear they are typically insufficient for host defense in the absence of CD4^+^ T cells (11). Macrophages become polarized to differential activation states by signals in the local tissue environment (12). These include T helper cytokines, microbial products, and growth factors. A growing body of research suggests that macrophage phenotype dictates the outcome of the Pc-macrophage interaction. Alternatively activated M2 macrophages are associated with Pc clearance (13) and possess anti-Pc effector function (14), which may represent a critical mechanism by which CD4^+^ T cells modulate macrophage function for effective host defense. A recent study found that AMs from Pc-susceptible immunosuppressed hosts adopt a classically activated M1 phenotype, while those from immunocompetent hosts adopt an M2 phenotype following Pc infection (15). These investigators also determined that the adoptive transfer of either M1 or M2 macrophages to Pc-infected immunosuppressed hosts enhanced Pc clearance, but less inflammation and better outcomes were observed following M2 transfer. However, another study found that neither IFNγ-dependent M1 polarization nor IL-4Rα-dependent M2 polarization is required for effective anti-Pc host defense (16). Thus many questions remain regarding the mechanisms governing the outcome of the macrophage-Pc interaction in the presence or absence of adaptive immunity.

AIDS patients, SIV-infected non-human primates, immunosuppressed rats, rabbits, and ferrets are all highly susceptible to Pc infection. Definitive studies in mice (17, 18) and rats (19) have shown that specific depletion of CD4^+^ T cells is sufficient to render these rodents susceptible to infection. The C57BL/6, Balb/c, and C.B-17 mouse strains are commonly used for studies of PcP and are highly susceptible when CD4-depleted or when bred onto a SCID or Rag background. Furthermore, several other inbred strains are also confirmed susceptible to PcP (20), and to our knowledge none have been reported as resistant to infection. In the current study we report that FVB/N mice (hereafter FVB) possess alveolar macrophages that are intrinsically programmed to provide protective innate immunity against *Pneumocystis* even in the absence of functional adaptive immunity. Although the specific factors responsible for FVB resistance are unknown, whole genome sequencing has identified genetic differences between FVB and other inbred strains such as C57BL/6 and Balb/c (21). FVB mice also express characteristic phenotypic variations relative to other inbred strains, including early onset retinal degeneration, C5 complement deficiency, and resistance to atherosclerosis and collagen-induced arthritis (21). Understanding the mechanisms of macrophage-mediated Pc resistance may help identify strategies to boost host defense and reduce PcP-related immunopathogenesis in susceptible populations.

## Materials and methods

### Ethics statement

All animal protocols used in this study were reviewed and approved by the University of Rochester's Committee on Animal Resources (UCAR; PHS Assurance number A-3292-01). The UCAR follows the Public Health Service (PHS) Policy on Humane Care and Use of Laboratory Animals, and is fully accredited by The Association for Assessment and Accreditation of Laboratory Animal Care (AAALAC).

### Mice

BALB/cJ (Stock #000651), C57BL/6J (Stock #000664), FVB (Stock #001800), and Nu/J (Stock #002019) mice were purchased from Jackson Laboratory (Bar Harbor, ME). C.B-*Igh1*^*b*^/IcrTac (C.B-17), C.B-*Igh-1*^*b*^/IcrTac-*Prkdc*^*scid*^ (C.B-17 SCID), and C.Cg/AnNTac-*Foxn1*^*nu*^ NE9 (BALB/c nude) mice were purchased from Taconic Farms, Inc. (Hudson, NY). Macrophages Insensitive to IFNγ (MIIG) mice were described previously (22). All mice were housed in the Animal Care Facility of University of Rochester Medical Center.

### Isolation of mouse *pneumocystis* organisms

*Pneumocystis* was isolated from lungs removed from heavily-infected C.B-17 SCID mice as previously described (23). The final preparation was stained with Gomori's methenamine silver to enumerate cysts, and Diff-Quick (Dade AG, Dudingen, Switzerland) to ensure no bacterial contamination was present. In addition, all preparations were plated on commercially available chocolate blood agar plates to test for the presence of other microorganisms. Experimental mice were infected with *Pneumocystis* organisms by direct intra-tracheal inoculation following isofluorane anesthesia, or by continuous co-housing of experimental mice with Pc-infected SCID mice.

### Depletion of T cell subsets

CD4^+^ and CD8^+^ T cells were depleted by intra-peritoneal administration of monoclonal antibodies every 4 days, as described previously (24, 25). Depletion protocols utilized 300 μg of anti-CD4 monoclonal antibody (clone GK1.5, American Type Culture Collection (ATCC), TIB207) or 250 μg of CD8^+^ T cell depleting MAb (clone 2.43, ATCC, TIB210). NK cells were depleted by intra-peritoneal administration of 250 μg of anti-NK1.1 monoclonal antibody (clone PK136, ATCC, HB-191™) every 3 days (26), and the immunosuppressive anti-CD3 antibody was given by intraperitoneal injection of 150 μg of F(ab')_2_ fragments of anti-CD3 (clone 145 2C11, ATCC, CRL-1975) every other day as previously described (27). To confirm that the anti-CD4, anti-CD8, and anti-NK antibodies effectively deplete the target cells in FVB mice, lung leukocytes and splenocytes were analyzed by flow cytometry using antibody clones distinct from those used for depletion. Antibodies used for flow cytometry were RM4-4 (anti-CD4), 53-6.7 (anti-CD8), and DX5 (anti-CD49b). The anti-CD4 and anti-CD8 antibodies depleted greater than 98% of the CD8^+^ cells from the lungs and spleens of FVB mice (Figure S1). Anti-NK antibody treatment depleted approximately 60% of the CD49b positive NK cells from the spleen and 55% of the CD49b positive NK cells from the lung (Figure S1). The lower depletion percentages for anti-NK1.1 depletion may be related to the presence of NK1.1 negative cells which are DX5 (CD49b) positive.

### Depletion of alveolar macrophages

Alveolar macrophage depletion was achieved by once weekly intra-tracheal administration of 80 μL of clodronate-liposomes (28, 29), and has been successfully used to study the role of alveolar macrophages in a variety of disease processes including *Pneumocystis* infection (30). Clodronate-liposomes or PBS-liposomes were synthesized at the University of Rochester School of Medicine and Dentistry according to published methods (31, 32). Control PBS-liposomes were prepared in an identical manner except that clodronate was omitted. After preparation, the liposomes were handled according to sterile procedure to minimize microbial contamination, and were used within 1 week of preparation. The amount of clodronate-liposomes used for AM depletion was determined empirically. A single dose of 80 μL of clodronate liposomes per mouse was found to result in an 80% reduction in the number of AMs, and a weekly instillation of this dose resulted in sustained AM depletion (Figure S2).

### Tissue processing and flow cytometry

Lungs were removed and minced with sterile scissors, and the tissue was passed through a 40 micron filter in 1 mL RPMI media. Five mL of RPMI containing 1 mg/ml collagenase (Sigma #C0130) and 0.086 mg/ml DNase I (Sigma #DN25) was added to the tissue, which was digested using the Miltenyi gentleMACS^TM^ Octo Dissociator. The tissue was digested at 37°C for 30 min and then passed through a fresh 40 micron filter. The cells were pelleted at 250xg for 10 min at 4°C. Red blood cells were lysed with RBC lysis buffer (Invitrogen) for 1 min, and then 9 ml of RPMI complete media was added. Cells were pelleted at 250xg for 10 min at 4°C, and resuspended in RPMI. For splenocyte recovery, spleens were pushed through a stainless steel strainer in 10 mL 1X HBSS with a syringe plunger. The cells were then passed sequentially through 70 micron and 40 micron filters. Cells were pelleted at 250xg for 10 min at 4°C, and washed with 1X HBSS. Isolated lung and spleen cells were washed with FACS buffer (1X PBS, 0.5% BSA, 0.05% sodium azide), incubated in Fc block (2.4G2, Tonbo Biosciences), and stained with CD4 (RM4-4, BD Pharmingen), CD8 (53-6.7, BD Pharmingen), CD3e (17A2, eBioscience), CD49b/Pan-NK cells (DX5, BD Pharmingen), CD45 (30-F11, eBioscience). For assessment of lung macrophage and monocyte populations, isolated lung cells were stained with anti-CD11c (BD Pharmingen), anti-F4/80 (BD Pharmingen), and anti-Ly6g (BD Pharmingen). 7AAD (Tonbo Biosciences) was used to exclude dead cells. Samples were analyzed on a FACS LSRII (Becton Dickinson) in the University of Rochester Medical Center Flow Cytometry Core facility and data was acquired using FlowJo v10 for Mac.

### Measurement of *pneumocystis* lung burden

*Pneumocystis* burden in the lungs of experimental mice was determined by quantitative real-time PCR (qPCR) as previously described (23). Briefly, the right lung lobes were homogenized in PBS (one ml of PBS per 150 mg of lung tissue) in a mechanical homogenizer. Homogenates were boiled for 15 min, vigorously vortexed for 2–3 min, and then centrifuged for 5 min at 12,000 x*g*. The supernatant was carefully removed and stored at −80°C for real-time qPCR analysis. Boiled samples were assayed by qPCR using TaqMan® primer/fluorogenic probe chemistry, and the CFX96™ Real-Time System (Bio-Rad Laboratories). A primer/probe set specific for a 96 nucleotide region of the mouse-derived *Pneumocystis* kexin gene (33) was designed using the Primer Express software (Applied Biosystems). The sequences of the primers and probe used were as follows: forward primer, 5'-GCACGCATTTATACTACGGATGTT-3'; reverse primer, 5'-GAGCTATAACGCCTGCTGCAA-3'; fluorogenic probe, 5'-CAGCACTGTACATTCTGGATCTTCTGCTTCC-3'. Quantitation was determined by extrapolation against standard curves constructed from serial dilutions of known copy numbers of plasmid DNA containing the target kexin sequence. Data were analyzed using the ABI Prism 7000 SDS v1.0 software (Applied Biosystems), and are reported as total kexin DNA copies per lung. *Pneumocystis* burdens were also confirmed microscopically by enumerating cysts with Gomori's methenamine silver (GMS) staining of lung homogenates (34).

### Determination of anti-*pneumocystis* antibody by elisa

Blood was collected from experimental mice by cardiac puncture, and the isolated sera were stored at −80°C. Levels of anti-*Pneumocystis* antibodies were determined by ELISA as described previously (35), with results expressed as absorbance at 405 nM.

### Adoptive transfer of alveolar macrophages

RAG2 knockout mice were depleted of resident AMs by intra-tracheal administration of a single dose of clodronate-liposomes 7 days before Pc infection. AMs were isolated from either Pc-susceptible (C.B-17) or Pc-resistant (FVB) mice by bronchoalveolar lavage. The AMs were washed with PBS and then directly instilled (in 100 uL PBS) into the lungs of RAG2 mice at 4 days (2e5 cells per mouse) and 3 days (5e5 cells per mouse) before Pc infection. A dose of 300 μg of CD4-depleting monoclonal antibody (clone GK1.5) was also administered intra-peritoneally at 4 days pre-infection, and again every week thereafter until the end of the experiment to ensure that no undetected CD4^+^ T cells were transferred with the AMs. After adoptive AM transfer, experimental mice were infected by intra-tracheal administration of 2e5 Pc cysts, and the Pc-burden in the lungs was then determined at 4 weeks post infection as described above.

### Real-time PCR quantitation of macrophage gene expression

FVB and CB.17 mice were CD4-depleted for 1 week as described above. Alveolar macrophages were isolated by bronchoalveolar lavage with 8 mLs of macrophage isolation buffer (Dulbecco's PBS, 1% glucose, 0.2 mM EGTA, 50 μg/mL gentamicin, pH 7.4). Recovered cells were centrifuged at 250 xg for 10 min, and resuspended in RPMI supplemented with 5% FBS, 200 mM L-glutamine, and 50 μg/mL gentamicin. Cells were counted and differential stain performed to enumerate macrophages. BAL cells were plated in 24 well tissue culture plates and incubated for 60 min at 37°C. Dishes were washed three times with media to remove any non-adherent cells, and the adherent macrophages were left untreated or stimulated with a 3:1 ratio of Pc:AM, 150 units/mL of recombinant murine IFNγ (Peprotech), or both for 6 h. Media was removed and cells were dissolved in TRIzol reagent (Invitrogen) for RNA isolation according to manufacturer's instructions. Reverse transcription of the RNA template was performed using the iScript cDNA synthesis kit (Bio-Rad), and real-time PCR quantitation was performed using the iQ™ SYBR® Green Supermix (Bio-Rad) according to manufacturer's instructions. RNA expression data for each was normalized to untreated AMs at 0 h. Bio-Rad PrimePCR primer pairs were used for mouse Arg-1 (qMmuCID0022400), YM-1 (qMmuCID0022826), Nos-2 (qMmuCID0023087), and Hprt (qMmuCID0005679). Other gene-specific primer pairs were designed using Primer-BLAST (NCBI) (36): MRC-1 forward 5′AGG CTG ATT ACG AGC AGT GG 3′ and MRC-1 reverse 5′ CCA TCA CTC CAG GTG AAC CC 3′; Dectin-1 forward 5′AGG CAT CCC AAA CTA CAG GAG 3′ and Dectin-1 reverse 5′TCA GTA GAT GAG CAC CTA GCT G3′; Fizz-1 forward 5′ ACT TCT TGC CAA TCC AGC TAA C 3′ and Fizz-1 reverse 5′ AGT AGC AGT CAT CCC AGC AG 3′; Mgl1 forward 5′GGCTCTCAAAACAGATCTGTC 3′ and Mgl1 reverse 5′AGCTGCCTTCATGCTCCG 3′. All primer pairs were designed to span an intron in the genomic sequence.

### Immunofluorescence staining

Freshly isolated alveolar macrophages (5e4) from uninfected mice were spun onto glass slides and fixed in 3% paraformaldehyde in PBS. The slides were rinsed PBS and permeabilized with 0.02% Triton X-100 in PBS. After washing, the slides were blocked with 5% normal goat serum in 0.05% Tween-20 in PBS (PBS-T). Slides were washed three times with PBS, and incubated in rabbit anti-mouse YM-1 antibody (STEMCELL Technologies, #01404) in 3% normal goat serum in PBS-T at 4°C overnight in a humidified chamber. Following three washes the slides were incubated for 1 h at room temperature in goat anti-rabbit IgG Alexa Fluor 488 (Invitrogen, #A11008) in 3% normal goat serum in PBS-T. Slides were washed, stained with DAPI (Molecular Probes), and coverslips were mounted with anti-fade Vectashield (Vector Laboratories). The mean fluorescent intensity of greater than 300 cells for each strain was quantified using ImageJ software (National Institutes of Health).

### IFNγ administration

Recombinant mouse IFNγ (2 μg per dose, Peprotech) was administered intra-tracheally on days−3 and−1 prior to infection with 5e5 freshly isolated mouse *Pneumocystis* organisms, and then every 3 days thereafter for the duration of the study.

### Statistical analyses

Sigma-Stat v. 3.5 (Systat Software Inc., San Jose, CA) was used for statistical analyses. Two groups were compared by *t*-test. The tests were considered statistically significant if *p* < 0.05. Multiple groups were tested by One Way Analysis Of Variance (ANOVA). If ANOVA was significant (*p* < 0.05), then individual groups were compared using the Student-Neuman-Keuls *post-hoc* test.

## Results

### FVB mice rapidly clear *pneumocystis* without triggering adaptive immunity

**Figure 1:**
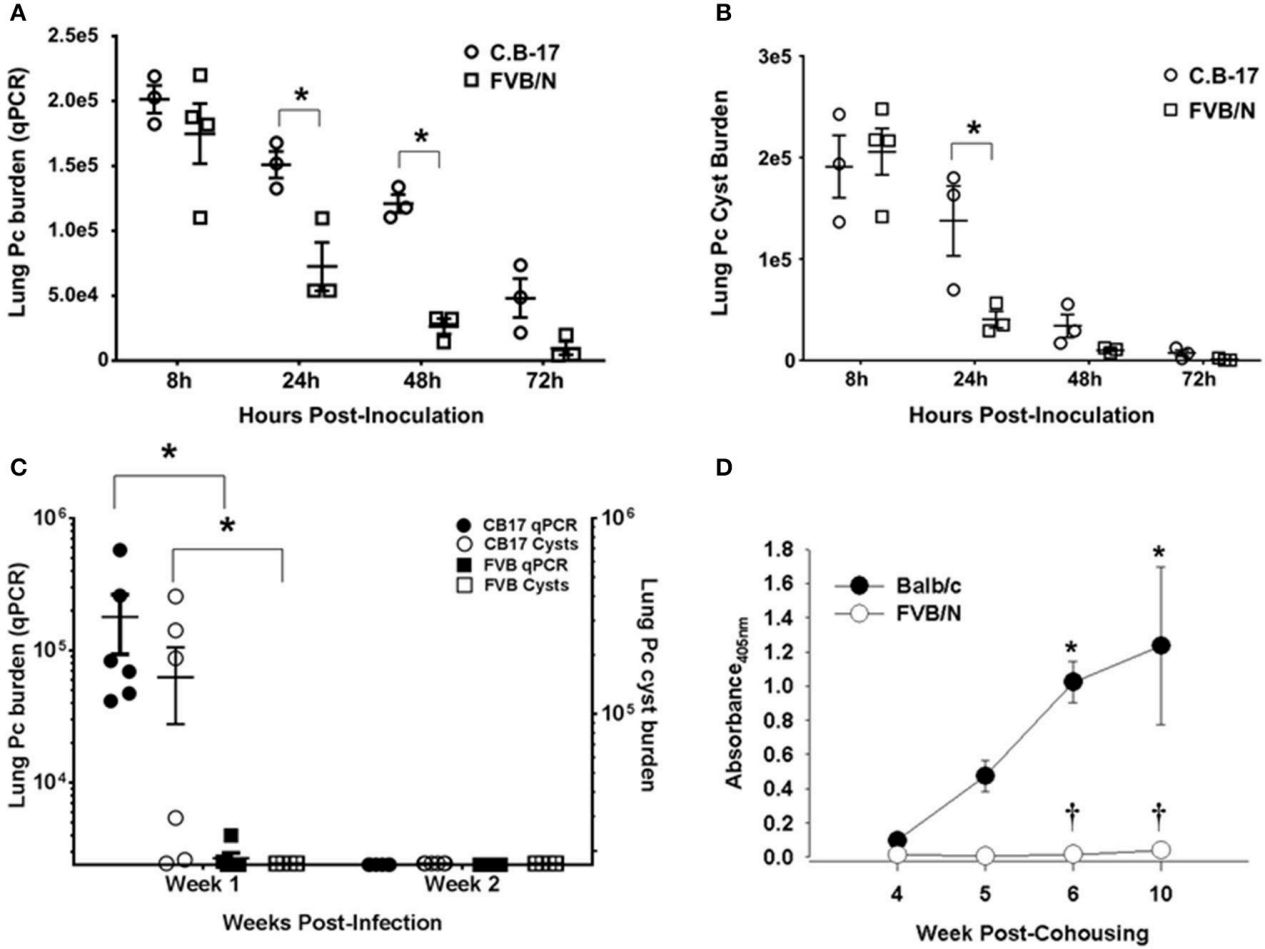
Rapid innate *Pneumocystis* clearance kinetics in FVB mice. Pc burden in the lungs of intra-tracheally infected mice was determined by quantitative real time PCR of the Pc *kexin* gene or Pc cyst counts in GMS stained lung homogenates. **(A,B)** Pc kexin copies and cyst count data, respectively, over a range of 8 to 72 h post-inoculation. The symbols represent data values for individual mice, and the horizontal lines and error bars show the mean ± 1 SEM. ^*^p < 0.05 as compared to C.B-17 at that time point. **(C)** The Pc burden in C.B-17 and FVB mice at 1 and 2 weeks post-inoculation is shown, with the symbol key given on the graphs. The symbols represent data values for individual mice, and the horizontal lines and error bars show the mean ± 1 standard error measurement (SEM) for each group (*n* = 6 per group). Statistically significant differences are designated as ^*^p < 0.05 as compared to C.B-17. **(D)** Anti-Pc ELISA antibody titers from the sera of experimental mice co-housed with infected SCID mice expressed as absorbance at 405 nm. Data are mean ± 1 SEM (*n* ≥ 4 mice at each time point). ^*^p < 0.05 compared to Balb/c at week 4 or *p* < 0.05 compared to Balb/c at a particular time point.

Immunocompetent mice of typical laboratory strains such as BALB/c, C.B-17, and C57BL/6 become transiently infected after exposure to *Pneumocystis*. Organisms proliferate in the lungs for a short period until a threshold level is reached and a CD4^+^ T cell-dependent adaptive immune response clears the infection (8, 9). To determine whether immunocompetent FVB mice become transiently infected, FVB and C.B-17 mice were infected with *Pneumocystis* by direct intra-tracheal inoculation. Lung fungal burden was measured at 8h, 24h, 48h, 72h, 1 week, and 2 weeks post-infection by quantitative real-time PCR and confirmed by cyst counts of silver stained lung homogenates. As shown in Figures 1A,B, FVB mice rapidly cleared the *Pneumocystis*, and organism burden was near the limit of detection by 48 h. In contrast, C.B-17 mice displayed slower initial clearance and the organisms persisted in the lung for at least 1 week before the adaptive immune response eradicated the infection. At 1 week post-infection the C.B-17 mice had a greater than 2-log higher organism lung burden than FVB mice (Figure 1C). Similar results were obtained when cysts were enumerated in the lung homogenates. Only C.B-17 mice had detectable cysts in the lung homogenates after 1 week, and the cysts were cleared by the second week (Figure 1C). Importantly, FVB mice had significantly lower qPCR values and very few detectable cysts at any time point after 24-h post-inoculation. These findings indicate that FVB mice are highly resistant to the transient *Pneumocystis* infection that is observed in other mouse strains.

Immunocompetent mice of Pc susceptible strains also become transiently infected when co-housed with heavily infected SCID mice. The fungal burden peaks at 4 to 5 weeks post-co-housing and is quickly cleared with a concomitant increase in anti-*Pneumocystis* antibody titers in the sera (8, 9). In order to determine whether immunocompetent FVB mice exposed to infected SCID mice reach the threshold level of infection required to trigger adaptive immunity and antibody production, Balb/c and FVB mice were continually co-housed with heavily infected SCID mice. *Pneumocystis*-specific antibody in the sera of exposed mice was measured at weeks 4 through 10 post-co-housing. As shown in Figure 1D, Balb/c mice mounted an antibody response to *Pneumocystis* that was readily detectable by week 4 and progressively increased through week 10. In contrast, FVB mice did not produce detectable amounts of anti-*Pneumocystis* antibody (Figure 1D). Failure to produce *Pneumocystis*-specific antibody demonstrates that FVB mice were able to rapidly clear inhaled fungal organisms through innate immune mechanisms that operated independently of adaptive immunity or antibody. Importantly, FVB mice were capable of producing anti-*Pneumocystis* antibody when immunized with a bolus of whole *Pneumocystis* by the intra-peritoneal or intra-tracheal route (Figure S3). Together, these data suggest that the innate immune system of FVB mice rapidly eradicates *Pneumocystis* from the lungs without triggering adaptive immunity.

### FVB mice display protective innate immunity against *pneumocystis* infection

**Figure 2:**
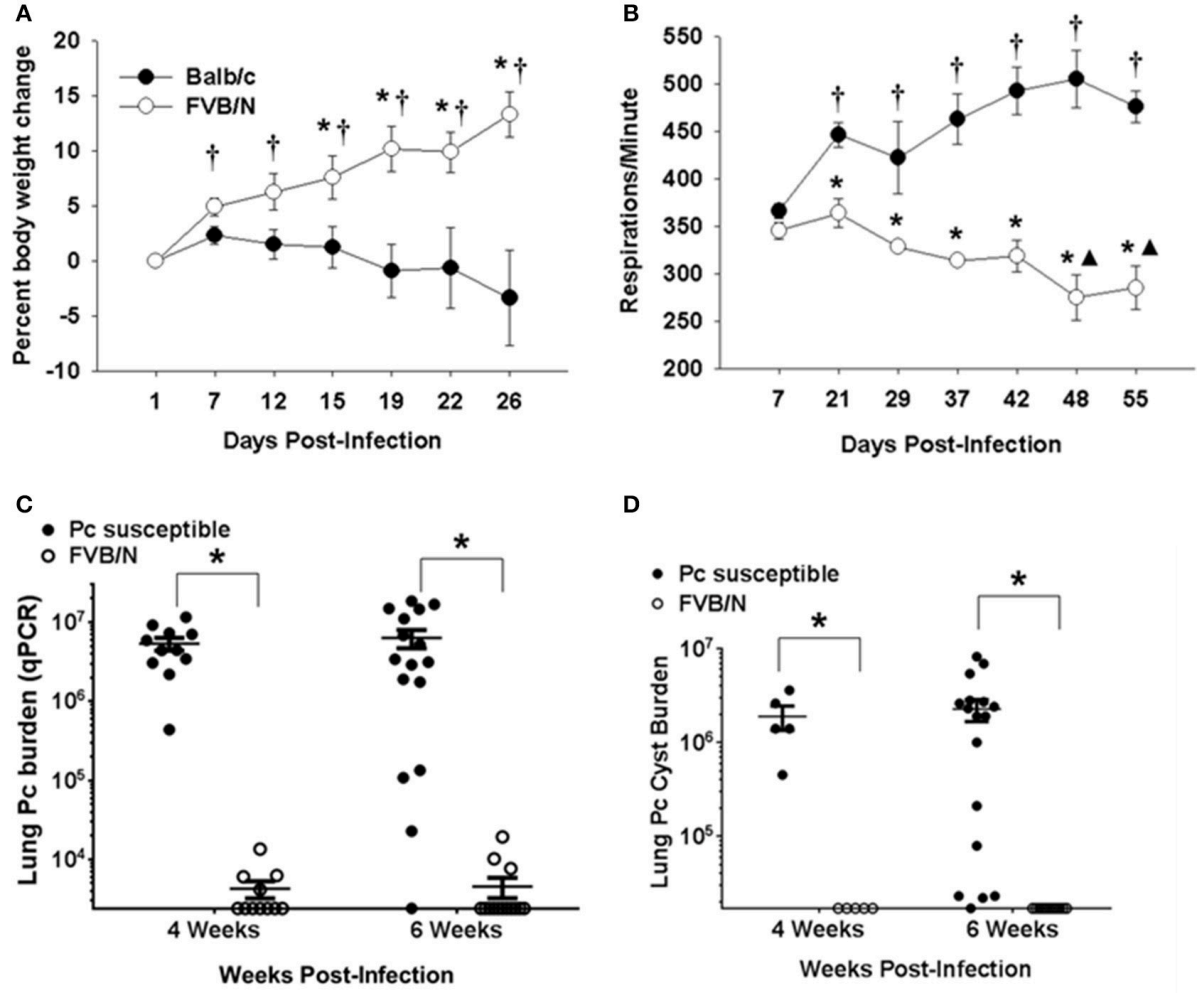
Innate protection against PcP in CD4-depleted FVB mice. Pc susceptible (Balb/c or C.B-17) and FVB mice were CD4 depleted and infected with Pc. **(A)** Percent body weight loss for experimental mice is plotted against the days post-infection. Each data point is the mean ± 1 SEM for a given mouse strain at the designated time (*n* = 6 mice per group at each time). Significant values are *p* < 0.05 compared to either the *mean weight change of the Balb/c group on that particular day or the mean weight change of the FVB group on day 1 post infection. **(B)** Changes in the respiratory rates (respirations per minute) are plotted against the days post-infection. Each data point is the mean ± 1 SEM for a given mouse strain at a designated time (*n* = 4 for Balb/c and *n* = 3 for FVB). Significant values are *p* < 0.05 compared to either the mean respiratory rate of Balb/c group on day 7, ^▴^the mean respiratory rate of FVB group on day 7, or ^*^the mean respiratory rate of Balb/c group on that particular day. **(C,D)** Pc lung burden as determined by quantitation of *Pneumocystis kexin* gene copies **(C)** or by Pc cyst counts **(D)** in experimental mice at 4 or 6 weeks post-infection. The symbols show data values for individual mice, and horizontal lines and error bars show the mean ± 1 SEM for each group (*n* ≥ 11 for qPCR data and *n* ≥ 5 for cyst data). Significant values are ^*^p < 0.05 for FVB mice compared to the Pc-susceptible group.

The results displayed in Figure 1 indicate that innate immunity limits *Pneumocystis* growth in immunocompetent FVB mice. To determine whether innate immunity was also sufficient to fully protect FVB mice from infection in the absence of CD4^+^ T cell mediated immunity, mouse strains known to develop severe PcP following CD4-depletion (Balb/c, C.B-17, or C57BL/6) and FVB mice were treated with anti-CD4 monoclonal antibody and infected via intratracheal inoculation. Flow cytometric analyses of splenocytes and lung leukocytes from CD4-depleted and non-depleted mice confirmed that anti-CD4 antibody successfully depleted greater than 98% of CD4^+^ T cells from the spleen and lungs of FVB mice (Figure S1). As expected, CD4-depleted Balb/c mice displayed characteristic signs of PcP, including progressive weight loss and elevated respiratory rates (Figures 2A,B). In contrast, FVB mice actually gained weight and showed no elevation in respiratory rate during the course of the study, suggesting that the mice did not develop active PcP. Lung *Pneumocystis* burden was enumerated by quantitative real time PCR (qPCR) of the single copy *kexin* gene, and by enumeration of Pc cysts in GMS stained lung homogenates (34). Pc-susceptible strains carried heavy fungal burdens at both 4 and 6 weeks post-infection (mean Pc *kexin* copies of 5.4 x 10^6^ and 6.3 x 10^6^, respectively; Figure 2C). In contrast, CD4-depleted FVB mice harbored very low Pc burdens at both time points, with values at the lower limit of detection of the assay (mean Pc *kexin* copies of 3.2 × 10^3^ and 2.7 × 10^3^, respectively). Enumeration of Pc cysts by microscopy yielded even more dramatic differences (Figure 2D). Pc cysts were readily detected in the lungs of the Pc-susceptible strains at 4 and 6 weeks post inoculation, while no cyst forms were found in the lungs of CD4-depleted FVB mice (Figure 2D). To further confirm that FVB mice harbored a very low level of infection, DNA samples from infected mice were amplified with primer pairs specific for the *Pneumocystis* gpA (37) or mitochondrial rRNA (38) genes using previously published PCR assays. Both of these genes are multi-copy in the Pc genome, and PCR–based assays to detect them are much more sensitive than those used to detect the single copy *kexin* gene (33). While strong bands corresponding to Pc gpA and mtRNA were amplified from the exposed Balb/c mice, no product was amplified for either gene from any of FVB samples tested (Figure S4). These results indicate either the complete absence of *Pneumocystis* or an extremely low level of infection in Pc-exposed FVB mice, and that CD4^+^ T cell independent mechanisms control Pc infection in FVB mice.

### Nu/J mice are resistant to *pneumocystis* infection

**Figure 3:**
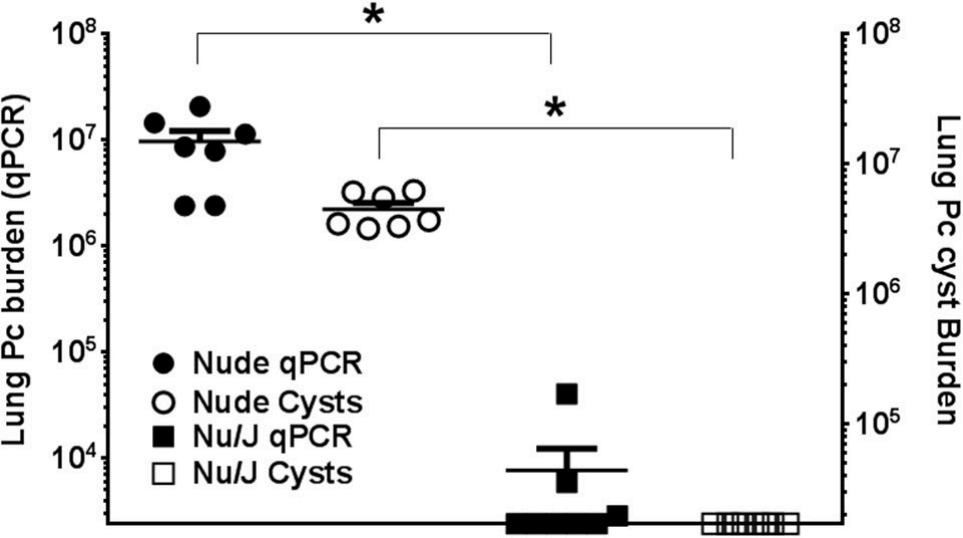
Comparison of Pc burden in nude mouse strains. Lung Pc burden in infected nude mouse strains 6 weeks after infection, as determined by qPCR of the Pc *kexin* gene **(**shaded symbols**)** or Pc cyst counts (open symbols) as determined by GMS stain. Symbols represent data values for individual mice. Horizontal lines and error bars represent mean ± 1 SEM for each mouse group (*n* ≥ 7). Balb/c nude: circles; Nu/J: squares. ^*^p < 0.05 compared to the Balb/c nude mouse group.

Nude mice lack mature T cells, and nude mice on a Balb/c background are highly susceptible to *Pneumocystis* infection (39, 40). However, the nude mutation is also present in the Nu/J strain that is very closely related to FVB. The FVB and Nu/J strains were both derived from a common ancestor strain (41), and we hypothesized that Nu/J mice would be protected from *Pneumocystis* infection by virtue of their strain background. Nude mice on a Balb/c background and Nu/J mice were infected with *Pneumocystis* by intra-tracheal inoculation, and then also continually co-housed with heavily infected SCID mice for 3 weeks after inoculation. As expected, the Balb/c nude mice developed severe *Pneumocystis* infection 6 weeks after infection, as evidenced by elevated respiratory rates and a blue hue to their hairless skin (data not shown). In contrast, the Nu/J mice did not appear ill, and their skin coloring remained normal. Assessment of fungal burden supported these observations. BALB/c nude mice harbored high fungal burdens as determined by both qPCR and cyst counts (Figure 3). In contrast, Pc cysts were not detected in the Nu/J mice, and only a few Nu/J mice had a very low number of detectable Pc *kexin* copies as determined by qPCR. These findings support and confirm that T cell-independent innate immunity is sufficient to protect FVB strain mice against infection by Pc.

### Neither T cells nor natural killer (NK) cells are required for protective innate immunity in FVB mice

**Figure 4:**
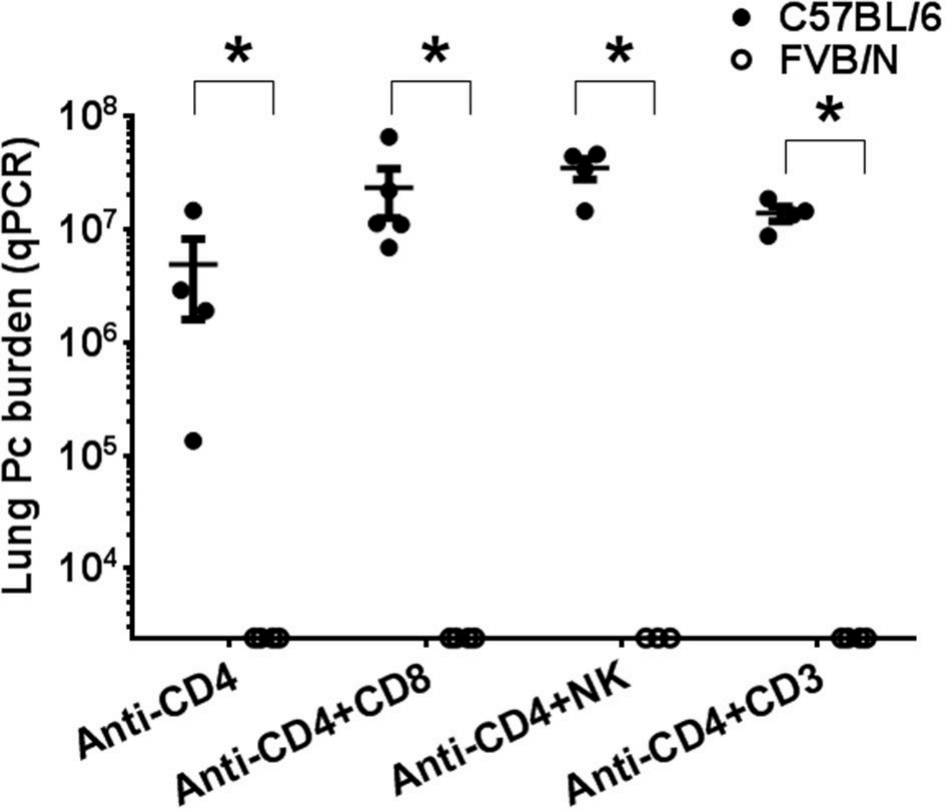
Comparison of *Pneumocystis* infection in mice depleted of immune cell subsets. CD4-depleted C57BL/6 (shaded circles) and FVB (open circles) mice treated with either anti-CD8, anti-NK1.1, or anti-CD3 antibodies were intra-tracheally inoculated with Pc organisms. Pc lung burden at 6 weeks post-infection was determined by qPCR of the Pc *kexin* gene. The symbols show data values for individual mice, and horizontal lines and error bars are mean ± 1 SEM for each group (*n* = 3–5). Statistically significant values are ^*^p < 0.05 compared to the C57BL/6 group receiving the same treatment.

To determine whether the mechanism of *Pneumocystis* resistance in FVB mice requires T cell subsets other than CD4^+^ T cells, multiple methods of T cell depletion were used. Recently it has been shown that NK cells limit *Pneumocystis* growth in CD4^+^ T cell-depleted hosts (42). In addition, a role for CD8^+^ T cell subsets in controlling *Pneumocystis* infection in the absence of CD4^+^ T cells has also been reported (43). To test the possibility that either CD8^+^ T cells, NK cells, or another population of CD3^+^ T cells was responsible for the innate resistance of FVB mice to *Pneumocystis*, susceptible C57BL/6 and resistant FVB mice were CD4-depleted and additionally treated with either anti-CD8 (clone TIB210), anti-NK (clone PK136) or anti-CD3 (clone 1452C11) antibody and then infected. For these studies C57BL/6J was chosen as the *Pneumocystis*-susceptible strain because mAb clone PK136 does not recognize nor deplete NK cells from BALB/c or C.B-17 mice (44). As expected, CD4-depleted C57BL/6 mice were susceptible to Pc infection, while CD4-depleted FVB mice remained resistant (Figure 4). Furthermore, CD4/CD8 double-depleted FVB mice remained resistant to *Pneumocystis* infection (Figure 4), demonstrating that CD8^+^ T cells are not required for the CD4^+^ T cell independent resistance of FVB mice. FVB mice depleted of both CD4^+^ and NK cells also remained resistant to infection. However it should be noted that NK cell depletion with anti-NK1.1, as assessed by CD49a staining (Figure S1), was not complete, and we cannot rule out the possibility that the remaining cells contribute to resistance. Immunosuppression of mice with anti-CD3^+^ monoclonal antibody (clone 1452C11) (45) impairs the function of all CD3^+^ T cells (46), and we have previously shown that mice treated with anti-CD3^+^ are not able to clear *Pneumocystis* from the lungs (27). As expected, C57BL/6 mice treated with anti-CD4 plus anti-CD3 antibodies became heavily infected. In contrast, FVB mice treated with anti-CD4 plus anti-CD3 antibodies remained resistant to *Pneumocystis* infection (Figure 4). These results confirm the results obtained using nude mice, and demonstrate that FVB resistance to Pc infection is not dependent upon T cell or NK cell effector function.

### Alveolar macrophages are required for resistance of FVB mice to *pneumocystis* infection

**Figure 5:**
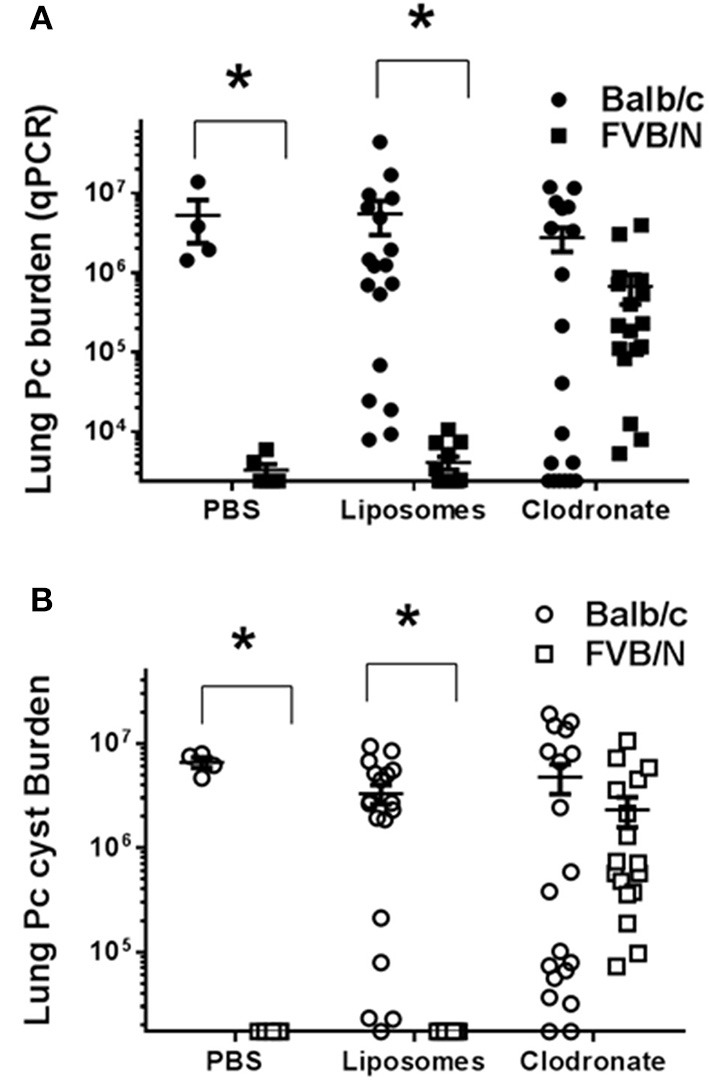
Comparison of Pneumocystis infection in mice depleted of alveolar macrophages. Lung Pc burden was determined by quantitative real time PCR of the Pc-specific *kexin* gene **(A)** or cyst counts as determined by GMS staining **(B)**. Balb/c (circles) or FVB/n mice (squares) were intra-tracheally instilled with either PBS, PBS-liposomes, or alveolar macrophage-depleting clodronate-liposomes, and then infected with *Pneumocystis*. Results shown are pooled from three independent experiments (see Table 1 for the results of each individual experiment). The symbols in the figure give values for individual mice in each group (*n* = 4–13 mice per group). Horizontal lines and error bars show the mean ± 1 SEM for the different groups, with statistical significance indicated as ^*^p < 0.05 for FVB/n mice compared to Balb/c mice for a given treatment.

Alveolar macrophages are key innate immune sentinel cells residing in the lung. While existing evidence suggests that AMs contribute to host defense against *Pneumocystis* (13, 30), they are typically insufficient for protection in the absence of CD4^+^ T cells (11). Chronic depletion of the alveolar macrophage population can be achieved by serial intra-tracheal administration of liposomes containing dichloromethylene diphosphonate (Cl_2_MDP-liposomes, or clodronate-liposomes) (47). This approach has been used to study the role of alveolar macrophages during pulmonary tuberculosis (48) and *Mycoplasma pulmonis* infections of mice (49). To determine whether alveolar macrophages are required for the CD4^+^ T cell-independent resistance of FVB mice to *Pneumocystis* infection, CD4-depleted Balb/c and FVB mice were depleted of AMs by intra-tracheal instillation of clodronate-liposomes. Control mice of each strain were inoculated with either PBS or PBS-containing liposomes. The efficiency of alveolar macrophage depletion with clodronate-liposomes was approximately 80% as determined by the differential staining of cells recovered by bronchoalveolar lavage and also by flow cytometry (Figure S2). Furthermore, clodronate-liposomes did not alter the number of lung interstitial macrophages or dendritic cells (Figure S2). As expected, CD4-depleted BALB/c mice treated with PBS, PBS-liposomes, or clodronate-liposomes developed high fungal lung burdens after infection as determined by qPCR (Figure 5A) and GMS staining of cysts (Figure 5B). In contrast, CD4-depleted FVB mice treated with PBS or PBS-liposomes remained resistant to infection. However, CD4-depleted FVB mice that were also depleted of AMs with clodronate-liposomes were rendered susceptible to *Pneumocystis* infection (Figures 5A,B). The results in Figure 5 are combined from three independent experiments, with the results of each individual experiment given in Table 1. In all experiments, FVB mice depleted of AMs became susceptible to Pc infection, while non-depleted FVB mice remained resistant (Table 1). These data demonstrate that alveolar macrophages are the critical innate immune effector cells that protect CD4-depleted FVB mice against *Pneumocystis* infection.

**Table 1:** Effect of Alveolar Macrophage depletion on Pc infection.

**Strain**	**Treatment**	**Experiment**	**Method of inoculation**	**# Of Infected/Total (%)**
FVB	Clodronate-Liposomes	I	Co-housing	7/8 (88%)
		II	I.T. Inoculation	10/10 (100%)
		III	I.T. Inoculation	7/7 (100%)
	None	I	Co-housing	0/8 (0%)
	PBS	II	I.T. Inoculation	0/3 (0%)
		III	I.T. Inoculation	0/3 (0%)
	PBS-Liposomes	II	I.T. Inoculation	0/3 (0%)
		III	I.T. Inoculation	0/3 (0%)
Balb/c	Clodronate-Liposomes	I	Co-housing	9/9 (100%)
		II	I.T. Inoculation	10/12 (83%)
		III	I.T. Inoculation	7/7 (100%)
	None	I	Co-housing	9/10 (90%)
	PBS	II	I.T. Inoculation	Not done
		III	I.T. Inoculation	4/4 (100%)
	PBS-Liposomes	II	I.T. Inoculation	11/11 (100%)
		III	I.T. Inoculation	7/7 (100%)

*Data from three independent experiments, with individual animals shown in pooled groups in Figure 5. Infected animals in the table were defined as positive if either the kexin copies and/or the cyst counts were equal to or greater than the limit of detection, i.e., 2 × 10^3^ or 1 × 10^4^ for kexin copies and cyst counts, respectively*.

### Adoptive transfer of FVB alveolar macrophages to RAG2^−/−^ mice protects against *pneumocystis* infection

**Figure 6:**
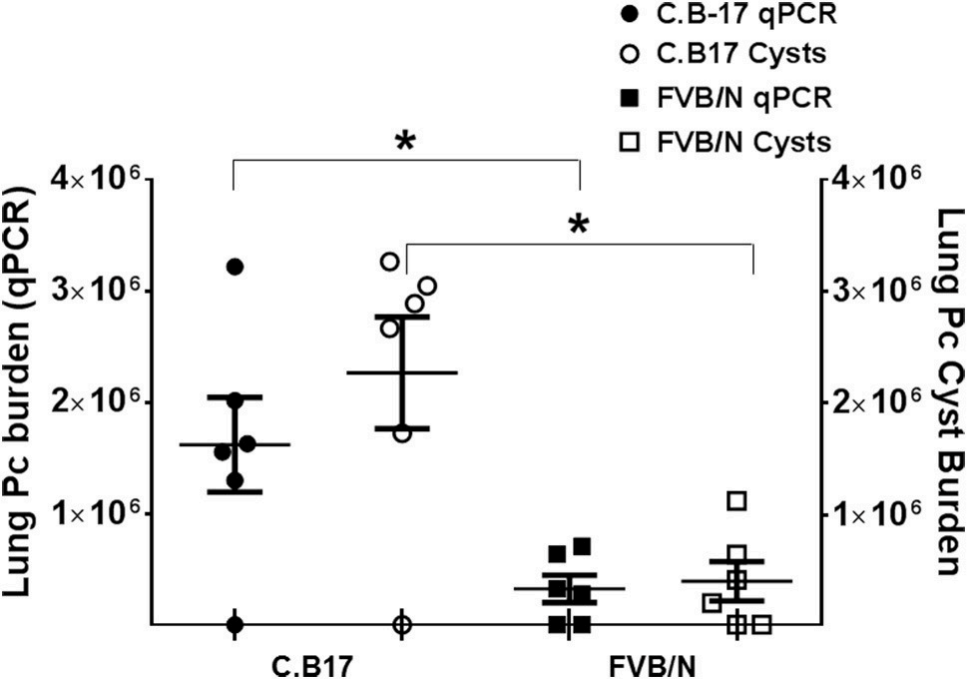
Pc burden in RAG2 mice reconstituted with alveolar macrophages from C.B17 or FVB mice. Freshly isolated AMs from either C.B17 or FVB mice were intra-tracheally instilled into RAG2 mice that had been depleted of endogenous AMs with clodronate. The RAG2 mice were then infected and Pc lung burdens were determined after 4 weeks by quantitative real time PCR of Pc-specific kexin gene (filled symbols) or by Pc cyst counts (clear symbols). The symbols represent data for individual mice, and horizontal lines and error bars show the mean ± 1 SEM of the different groups (*n* = 6). ^*^p < 0.05 for FVB AM recipient mice compared to C.B17 AM recipient mice.

If FVB alveolar macrophages are inherently more efficient for eradicating *Pneumocystis* than those of the susceptible strains, then adoptive transfer of FVB AMs to susceptible mice should result in enhanced host defense against infection. To test this, freshly isolated C.B-17 or FVB AMs were adoptively transferred to RAG2 mice by direct intra-tracheal instillation. The mice were then inoculated with Pc and sacrificed at 4 weeks post-infection, at which time Pc lung burden was measured by qPCR and GMS staining. The results are summarized in Figure 6. As shown, RAG2 mice that received AMs from *Pneumocystis*-resistant FVB mice had an approximately 5-fold lower Pc burden in their lungs compared to RAG2 mice that received AMs from the susceptible C.B-17 strain. This result supports our prior finding that FVB alveolar macrophages possess direct anti-*Pneumocystis* effector activity, and also demonstrates that the transfer of FVB AMs to susceptible mice can enhance CD4^+^ T cell-independent anti-*Pneumocystis* host defense.

### The *pneumocystis* resistant phenotype of FVB mice is a genetically dominant trait

**Figure 7:**
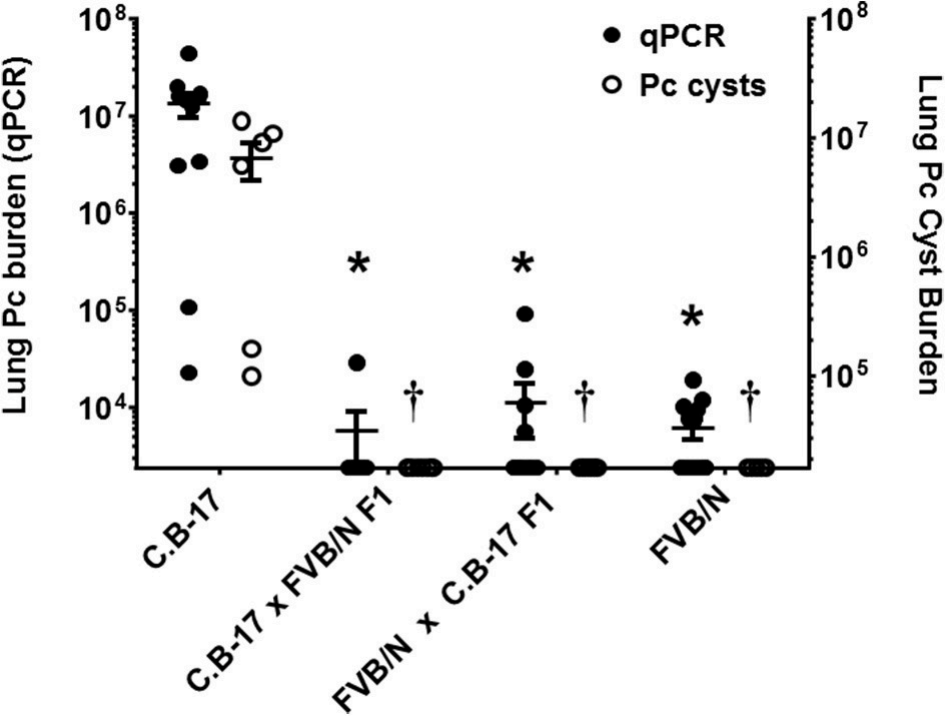
Susceptibility of hybrid mice generated by crossing resistant and susceptible mice. CD4-depleted mice of designated strains were infected and lung Pc burden at 5 weeks post-infection was determined by qPCR of the Pc *kexin* gene (filled circles and left axis) or Pc cysts counts by GMS stain (open circles and right axis). To generate crosses, either male C.B-17 mice were bred with female FVB mice (C.B-17 × FVB), or male FVB mice were bred with female C.B-17 mice (FVBN × C.B-17). All hybrid mice were resistant to *Pneumocystis* infection. The symbols show data values for individual mice, and the horizontal lines and error bars show mean ± 1 SEM for the different groups (*n* ≥ 8). ^*^p < 0.05 compared to qPCR mean of susceptible strain; *p* < 0.05, compared to the cyst count mean of the susceptible strain.

The strain-specific *Pneumocystis* resistant phenotype of FVB mice is likely determined by a gene or a set of genes preserved in this inbred strain. In order to determine whether resistance is a genetically dominant or recessive trait, F1 hybrids were generated by crossing susceptible C.B-17 mice with resistant FVB mice. Crosses of male C.B-17 with female FVB (C.B-17 × FVB) and vice-versa (FVBN × C.B-17) were included. Hybrid mice were CD4-depleted and then infected with *Pneumocystis*. Susceptibility was determined by qPCR and GMS staining as described. As shown in Figure 7, all of the hybrid mice were highly resistant to *Pneumocystis* infection. These data indicate that the phenotypic traits sufficient for FVB resistance to *Pneumocystis* are expressed in F1 hybrid crosses of resistant and susceptible mice.

### IFNγ administration directly reprograms resistant macrophages to a *pneumocystis* susceptible phenotype

**Figure 8:**
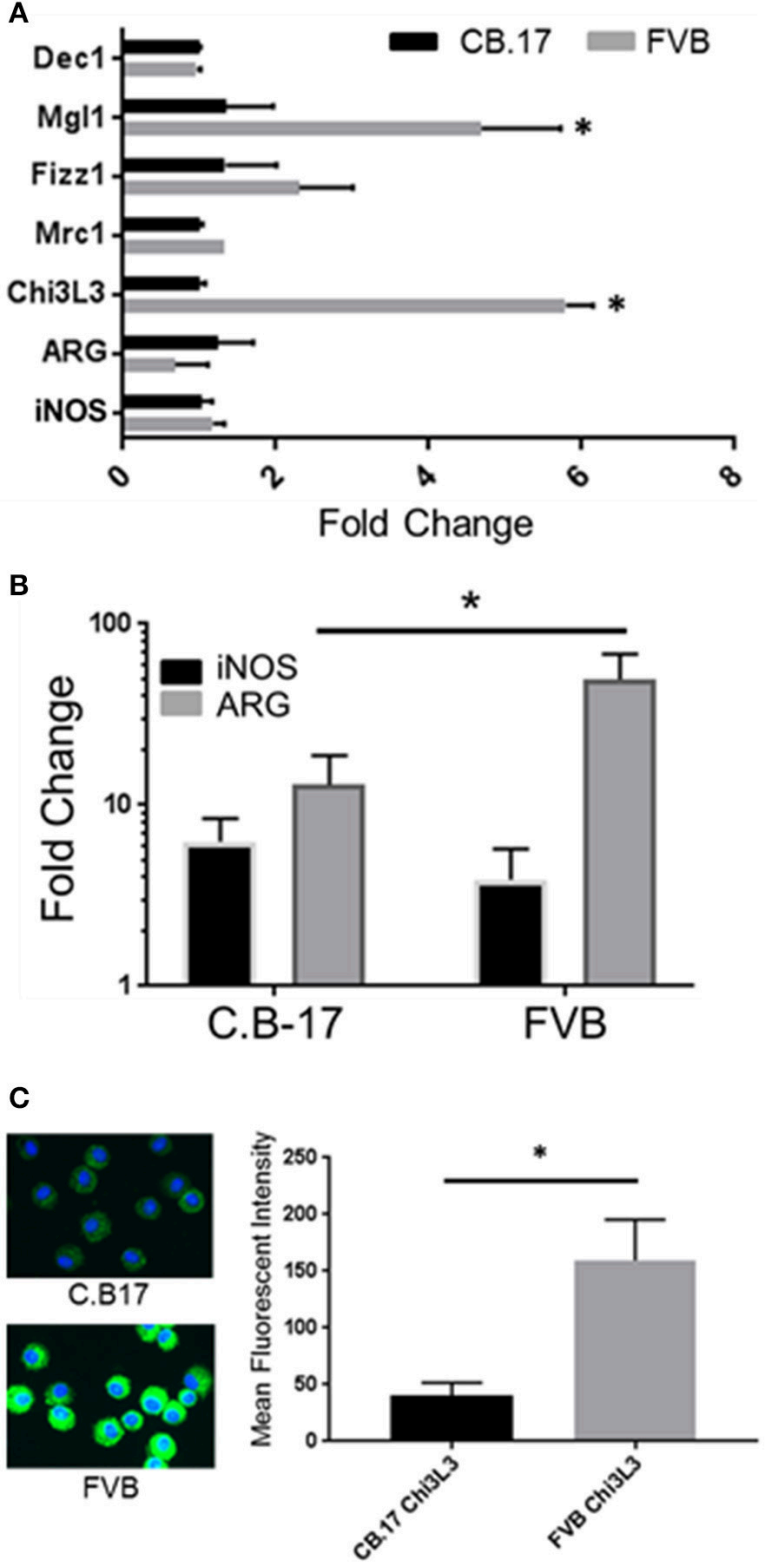
FVB alveolar macrophages display markers of M2 bias. Alveolar macrophages were obtained from FVB or C.B-17 mice by bronchoalveolar lavage. Total RNA was isolated from unstimulated AMs **(A)** or AMs stimulated for 6 h with freshly isolated *Pneumocystis* **(B)**. Reverse transcriptase real-time PCR was used to quantify the relative expression of M1 and M2 associated markers. Greater expression of the M2 marker CHI3L3 in FVB macrophages was confirmed at the protein level by immunofluorescence staining of freshly isolated C.B-17 and FVB AMs **(C)**. The bars represent the mean ± 1 SEM of fold change in gene expression in **(A,B)** (*n* = 3–6). The bars represent the mean fluorescent intensity ± 1 SEM in **(C)** (*n* > 300 cells). ^*^p < 0.05 for FVB AMs compared to C.B17 AMs.

**Figure 9:**
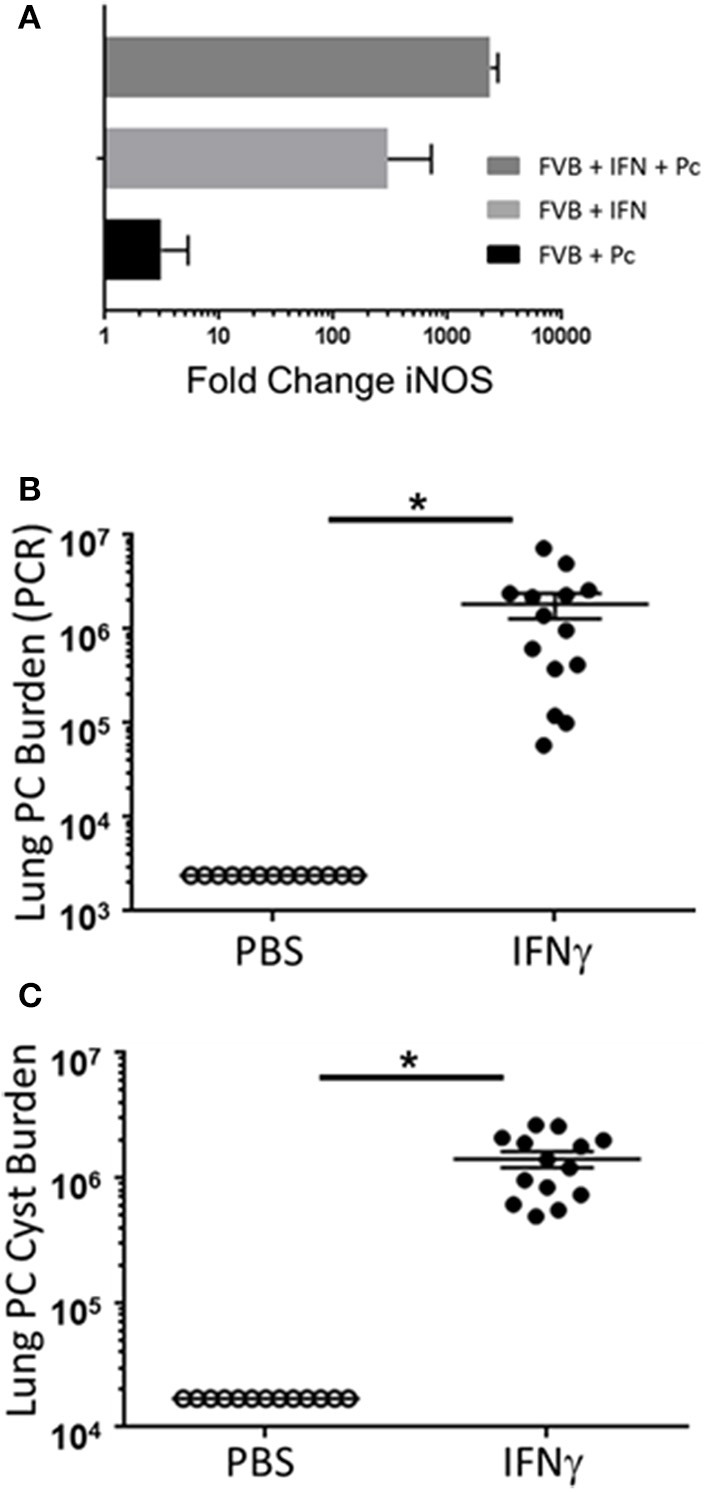
The Th1 cytokine IFNγ reprograms resistant FVB mice to a susceptible phenotype. Alveolar macrophages from FVB mice were cultured with IFNγ, Pc, or both for 6 h. Total RNA was isolated and reverse transcriptase real-time PCR was used to quantify the relative expression of the M1 marker iNOS **(A)**. The bars represent the fold change in gene expression compared to control untreated FVB alveolar macrophages (*n* = 6). CD4-depleted FVB mice were administered 2 μg of recombinant IFNγ or PBS vehicle via tracheal instillation on days −3 and −1 prior to infection with 5e5 mouse *Pneumocystis* organisms, and then every 3 days thereafter for the duration of the study. Lung Pc burden was measured at 5 weeks post-infection by qPCR of the Pc *kexin* gene **(B)**, and by Pc cyst counts of GMS stained lung homogenates **(C)**. Symbols represent data values for individual mice. Horizontal lines and error bars represent mean ± 1 SEM for each mouse group (*n* ≥ 13 per group). PBS treated mice: open circles; IFNγ treated mice: closed circles. ^*^p < 0.05.

**Figure 10:**
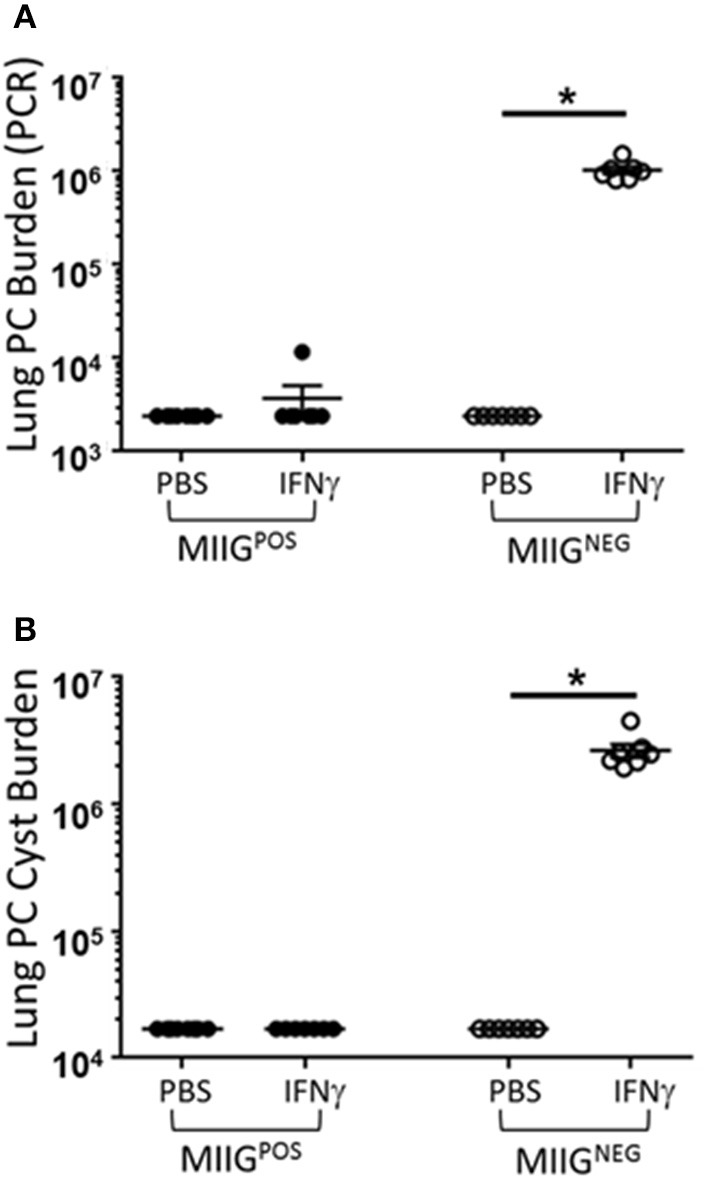
IFNγ directly reprograms resistant FVB macrophages to a Pc susceptible phenotype. FVB WT mice were crossed with C57BL/6 MIIG^+/−^ transgenic mice. MIIG positive and MIIG negative (WT) mice were identified and CD4-depleted. Groups of MIIG positive and MIIG negative mice were administered either 2 μg of recombinant IFNγ or PBS vehicle via tracheal instillation on days −3 and −1 prior to infection with 5e5 mouse *Pneumocystis* organisms, and then every 3 days thereafter for the duration of the study. Lung Pc burden was measured at 5 weeks post-infection by qPCR of the Pc *kexin* gene **(A)**, and by Pc cyst counts of GMS stained lung homogenates **(B)**. Symbols represent data values for individual mice. Horizontal lines and error bars represent mean ± 1 SEM for each mouse group (*n* ≥ 7 per group). MIIG negative mice: open circles; MIIG positive mice: closed circles. ^*^p < 0.05.

Prior studies have indicated that FVB mice have a Th2/M2 immune bias (50). To determine whether FVB alveolar macrophages possess characteristics of M2 macrophages at baseline, mRNA expression of a panel of M2 markers were evaluated. FVB AMs demonstrated significantly higher expression of the M2 markers CHI3L3 and MGL1 at baseline than AMs from the susceptible C.B-17 strain (Figure 8A). FIZZ1 expression was also higher in FVB AMs, but this trend did not reach statistical significance. In addition, co-culture of AMs with *Pneumocystis* produced a differential shift in the ratio of arginase to iNOS mRNA expression, with FVB AMs displaying a significantly greater increase in arginase expression than C.B-17 AMs (Figure 8B). In contrast, C.B-17 AMs showed a trend toward greater iNOS expression than FVB AMs following co-culture with *Pneumocystis*. Confirmation of an M2 bias in FVB AMs was demonstrated at the protein level by staining primary AMs with antibody to the prototypical M2 marker CHI3L3. Freshly isolated FVB AMs displayed a more than 3-fold greater mean fluorescent intensity than CB.17 AMs (Figure 8C). These data demonstrate that FVB AMs are biased toward an M2 phenotype at baseline, and are even more strongly polarized to an M2 phenotype following the interaction with *Pneumocystis*. Therefore, to determine whether shifting the intrinsic programming of FVB macrophages to an M1 biased phenotype would disrupt protective innate resistance to *Pneumocystis*, FVB mice were treated with recombinant mouse IFNγ via direct tracheal administration. FVB AMs treated with IFNγ demonstrated a dramatic increase in iNOS mRNA expression which was potentiated by *Pneumocystis* stimulation, demonstrating that they are capable of adopting an M1 polarized phenotype (Figure 9A). Alveolar macrophages recovered from IFNγ-treated FVB mice also displayed robust MHCII expression, another hallmark of M1 activation (Figure S5). Infectious challenge of IFNγ-treated CD4-depleted FVB mice with *Pneumocystis* demonstrated that they were highly susceptible to infection (Figures 9B,C). To further define the mechanism by which IFNγ impairs protective innate FVB resistance against Pc, macrophages insensitive to IFNγ (MIIG) mice were used (22). These mice express a dominant negative transgene from a macrophage-specific promoter, which renders macrophage populations unable to respond to IFNγ. As detailed above, when FVB mice are crossed with a susceptible mouse strain all offspring are resistant to *Pneumocystis* infection. Thus FVB mice were crossed with MIIG mice and the offspring were genotyped for the MIIG transgene. Transgene negative littermates were used as wild type controls. All MIIG positive and negative offspring were resistant to *Pneumocystis* infection when CD4-depleted and infected (Figure 10), demonstrating that macrophage IFNγR signaling was not required for the observed FVB resistance to *Pneumocystis*. As expected, transgene negative offspring (which respond normally to IFNγ) were rendered susceptible following IFNγ treatment. In contrast, all MIIG transgene positive mice, which possessed macrophages that were insensitive to IFNγ, remained resistant to infection following IFNγ treatment (Figure 10). These data demonstrate that unlike common susceptible laboratory strains, FVB mice possess alveolar macrophages that are programmed for innate *Pneumocystis* resistance. Furthermore, FVB alveolar macrophages are directly reprogrammed to a *Pneumocystis*-susceptible phenotype by the Th1 cytokine IFNγ.

## Discussion

The key immune cells required for host defense against *Pneumocystis* are CD4^+^ T cells (17), and humans, non-human primates, rats, ferrets, and many inbred strains of mice are all highly susceptible to *Pneumocystis* when CD4^+^ T cell function is impaired. Macrophages are also critical effector cells for the removal of *Pneumocystis* from the lung during a protective adaptive immune response (13). However, despite the fact that these tissue resident cells express pattern recognition molecules capable of recognizing antigenic and structural components of *Pneumocystis*, they have thus far proven incapable of providing effective host defense in the absence of CD4^+^ T cell helper function (11). In the current manuscript we report that alveolar macrophages from FVB mice are intrinsically programmed to provide protective innate immunity against *Pneumocystis* infection. Our experiments specifically verified that the ability of FVB mice to resist infection with *Pneumocystis* did not depend on specific T-cell populations that might have compensated for the loss of CD4^+^ T cells. These antibody-mediated depletion CD8^+^ T cells or NK cells in addition to CD4-depletion did not render FVB mice susceptible to PcP. Furthermore, disruption of CD3^+^ T cell function with an anti-CD3 antibody also did not impair FVB resistance.

Although T cells were not required for FVB resistance, the chronic depletion of alveolar macrophages did render FVB mice susceptible to infection, indicating that FVB alveolar macrophages are responsible for FVB innate resistance to PcP. This finding was in sharp contrast to the inability of alveolar macrophages from other mouse strains to provide innate protection against *Pneumocystis*. Our additional finding that anti-*Pneumocystis* antibody could not be detected in the sera of immunocompetent FVB mice upon continual co-housing with *Pneumocystis*-infected SCID mice suggests that FVB alveolar macrophages were able to clear infectious *Pneumocystis* organisms before they reached the required threshold to trigger an adaptive immune response. The importance of an enhanced early macrophage-*Pneumocystis* interaction in FVB mice was further supported by the observation that we failed to detect any organisms in the lungs of immunocompetent FVB mice just 1 week after Pc-inoculation, whereas Pc was readily detectable in the lungs of C.B-17 mice at that time. The rapid clearance of *Pneumocystis* without eliciting an adaptive pulmonary immune response in FVB mice is of particular interest because of the major role that immunopathogenesis plays in PcP-related lung injury.

Our findings support the interpretation that alveolar macrophages from FVB mice are either inherently activated for *Pneumocystis* clearance, or are programmed to transition to a protective activation state upon exposure to *Pneumocystis*. These results are also consistent with prior studies by Knott et al (51) that have reported that FVB mice are resistant to primary infection by the nematode *Nippostrongylus brasiliensis*. These authors concluded that the resistance of FVB mice was likely due to innate rather than adaptive immune mechanisms, and suggested that the critical damage to the parasite occurred during the lung stage of the infectious process. Importantly, M2 macrophages have been found to provide transferable protection against *Nippostrongylus* to the lungs of mice, indicating that an M2 phenotype is protective against the lung stage of this parasite (52). FVB mice exhibit a Th2 bias during immune and allergic responses, and we determined that alveolar macrophages from FVB mice express markers of alternative activation. Thus, it seems likely that the M2 macrophage bias in the FVB lung that protects against *Nippostrongylus* also protects against *Pneumocystis* infection.

Prior studies have found that M2 biased macrophages have effector activity for the clearance of *Pneumocystis* from the lung. For example, the treatment of mice with the immunomodulatory drug sulfasalazine (SSZ) accelerated Pc clearance in an immune reconstitution model of PcP (13). Augmented clearance was strongly associated with an alternatively-activated M2 macrophage signature. Similarly, Nelson et al. (14) have shown that macrophage polarization to a M2 phenotype enhances *Pneumocystis* killing, and Nandakumar et al. reported that M2 macrophages are associated with successful host defense in immunocompetent rats (15). However, our attempts to impair FVB resistance using IL-4Rα blocking antibodies were unsuccessful (data not shown), which is consistent with our prior studies showing that macrophage IL-4Rα is not absolutely required for normal CD4-dependent clearance of *Pneumocystis* from susceptible strains (16). Therefore we modified our approach from blocking M2 activation to promoting M1 activation by administering IFNγ. Exogenous IFNγ significantly impaired FVB innate resistance, and rendered these normally resistant mice highly susceptible to *Pneumocystis* infection. The innate immune dampening effects of IFNγ required direct interaction with the macrophage IFNγR, leading to speculation that M1 biased macrophages are permissive for Pc infection in immunodeficient mice. Importantly, these findings highlight the differences between the host defense requirements for *Pneumocystis* and other pathogenic fungi. IFNγ plays an important role in host defense against several fungal infections, and exogenous IFNγ has been proposed as an adjunctive therapy to accelerate fungal clearance (53–56). The identification of a mouse strain possessing a resistant alveolar macrophage population will provide a platform to compare and contrast these macrophages to identify important mechanisms of innate resistance to *Pneumocystis*, and to also understand the mechanisms utilized by *Pneumocystis* to evade innate immunity and persist as an opportunistic pathogen.

The finding that alveolar macrophages are capable of protective host defense against Pc infection in the absence of functional adaptive immunity could have important ramifications for the development of new strategies for the prophylaxis and treatment of PjP. Macrophages are key multifunctional effector cells that are able to adopt distinct phenotypes that correlate with specified and diverse functions related to host defense, inflammation, and tissue repair. Cytokines and growth factors, microbial products, and various classes of drugs are all able to modify macrophage phenotype and function. Our study suggests that FVB alveolar macrophages express markers of alternative M2 activation, which are associated with protective innate immunity against Pc. Importantly, FVB AMs can be pushed to a permissive phenotype by the M1 promoting cytokine IFNγ. A large number of studies over the past several years have focused on understanding the mechanisms regulating macrophage phenotype, and how these various phenotypes function in health and disease (12). As the required mechanisms by which FVB AMs protect against Pc are defined, this insight may be used to identify modulatory strategies to enhance macrophage-mediated innate immunity in immunosuppressed patients. Modulation of macrophage phenotype could also be developed as a treatment option to enhance Pc clearance while dampening the Pc-driven immune response that is a major component of PcP-related pathogenesis. It is noteworthy that alternatively activated phenotypes, which promote Pc killing, also possess anti-inflammatory and reparative functions. Thus, promoting alternative macrophage activation could have dual benefit for improving patient outcome. Recent retrospective patient studies have expanded upon our finding that sulfasalazine promotes an M2 macrophage phenotype and enhances Pc clearance. These investigators utilized a large cohort of rheumatoid arthritis patients to demonstrate that sulfasalazine use may have prophylactic value against the development *Pneumocystis jirovecii* pneumonia (57). Other agents currently used clinically, including the antibiotic azithromycin, have also been found to promote alternative macrophage activation (58, 59).

In summary, the current study has demonstrated that FVB mice are protected against PcP even in the absence of functional adaptive immunity, which is typically required for mammalian host defense against this fungal pathogen. The mechanism of innate resistance was shown to be alveolar macrophage-dependent, and is currently being explored in more detail. The FVB model of innate resistance provides an important tool to identify the specific mechanisms that dictate the outcome of the Pc-macrophage interaction. This study also raises the possibility that appropriate modulation of macrophage phenotype and function could offer a novel therapeutic approach to treat and/or prevent respiratory fungal infections.

## Author contributions

SB (major), TW (major), FG, ME, RN, PM, MJ, and JW contributed to the conception or design of aspects of the study. SB, JW, PM, FR-E, JM, and ZW contributed to the acquisition and analyses of the data. SB (major), TW (major), FG, ME, PM, FR-E, and RN contributed to interpretation of the data. TW, SB, FG, RN, ME, and ZW wrote portions of the manuscript. All authors contributed to manuscript revision, read and approved the submitted version.

### Conflict of interest statement

The authors declare that the research was conducted in the absence of any commercial or financial relationships that could be construed as a potential conflict of interest. The reviewer LS and handling Editor declared their shared affiliation.
